# Effects of zoledronic acid therapy in fibrous dysplasia of bone: a single-center experience

**DOI:** 10.20945/2359-3997000000459

**Published:** 2022-04-19

**Authors:** Luciana Pinto Valadares, Bruno Silva de Araújo Ferreira, Bernardo Matos da Cunha, Larissa Aniceto Moreira, Frederico Gideoni Albinati Batista, Cristiane da Fonseca Hottz, Gabriel Galvão Rafael Magalhães

**Affiliations:** 1 Hospitais de Reabilitação Brasília DF Brasil Ambulatório de Osteometabolismo, Rede SARAH de Hospitais de Reabilitação, Brasília, DF, Brasil; 2 Universidade do Planalto Central Apparecido dos Santos Distrito Federal Brasil Universidade do Planalto Central Apparecido dos Santos (UNICEPLAC), Gama, Distrito Federal, Brasil

**Keywords:** Fibrous dysplasia, McCune-Albright syndrome, zoledronic acid, bisphosphonate therapy

## Abstract

**Objective:**

Fibrous dysplasia (FD) is a rare bone disorder that can involve any part of the skeleton, leading to bone pain, deformities, and fractures. Treatment with intravenous bisphosphonates has been used with variable results. Therefore, we aimed to evaluate the effects of zoledronic acid (ZA) therapy in patients with monostotic or polyostotic FD.

**Subjects and methods:**

The medical records of thirteen patients with FD evaluated between 2015 and 2020 were retrospectively analyzed. In the subgroup of patients treated with ZA (n = 7), data on pain relief, changes in bone turnover markers (BTMs), and adverse events following ZA infusions were retrieved. Moreover, radiological changes in response to treatment were recorded in patients who underwent radiological follow-up.

**Results:**

Of the patients, 5 (38%) presented with monostotic whereas 8 (62%) had polyostotic FD. Bone pain was a common finding (69%), and most patients (62%) exhibited elevated baseline BTMs. Partial or complete pain relief was reported in 6 of 7 patients treated with ZA. BTMs, especially C-telopeptide of type I collagen (CTX), significantly decreased after therapy (change rate: −61.8% [IQR −71, −60%]), and median CTX levels were significantly lower than at baseline (0.296 ng/mL [0.216, 0.298] vs. 0.742 ng/mL [0.549, 0.907], respectively; P = 0.04). No radiological improvement was observed in cases with radiological follow-up (n = 3). No serious adverse effects of ZA were reported.

**Conclusion:**

ZA treatment was well tolerated and provided beneficial effects in relieving bone pain and reducing BTMs, especially CTX. Our data reinforce the role of ZA in the treatment of FD-related bone pain.

## INTRODUCTION

Fibrous dysplasia (FD) is a rare mosaic bone disorder caused by somatic activating mutations in *GNAS* , which encodes the Gαs subunit of G-coupled protein receptors. In bone, constitutive Gαs activation impairs the differentiation of skeletal stem cells, leading to the replacement of normal bone and marrow with immature woven bone and fibrotic stroma ( 1 – 3 ). Additionally, there is evidence of active osteoclastogenesis and increased bone resorption in dysplastic bone ( 3 , 4 ).

Any part of the skeleton can be affected, and the clinical spectrum ranges from an asymptomatic monostotic lesion discovered incidentally to severely disabling polyostotic disease. FD may occur alone or in association with extraskeletal features, such as café-au-lait skin macules and hyperfunctioning endocrinopathies, which is termed McCune-Albright syndrome (MAS) ( 1 , 3 ). Complications of bone involvement include bone pain; skeletal deformities; fractures; functional impairment; and neurological complications, such as visual disturbances and/or hearing impairment, due to nerve entrapment. Malignant sarcomatous degeneration rarely occurs ( 1 , 2 ).

Antiresorptive agents, such as bisphosphonates, have emerged as potential treatment options for FD due to high osteoclastic activity in dysplastic bones. Intravenous bisphosphonates, such as pamidronate and zoledronic acid (ZA), have been used to treat patients with FD and can provide pain relief and a reduction in bone turnover markers (BTMs), but their potential effects on disease progression are unclear ( 5 ). In an open study of 58 patients treated with pamidronate bone pain significantly improved, and BTMs markedly decreased after treatment. In addition, about 50% of patients had radiological improvement of bone lesions, evidenced by filling of osteolytic lesions and/or cortical thickening ( 6 ). In another study of 22 patients with polyostotic FD/MAS treated with bisphosphonates, bone pain was alleviated in 64% of patients. The alkaline phosphatase (ALP) levels decreased in most patients after pamidronate or ZA therapy, but radiological effects were not evaluated ( 7 ). Recently, Chapurlat and Legrand reviewed the studies published up to the end of 2019 evaluating the effects of bisphosphonates treatment in FD. Overall, data from the selected studies showed intravenous bisphosphonate therapy decreased bone pain and bone resorption markers in most patients, but improvement of radiological lesions was an inconsistent finding ( 8 ). However, available data of intravenous bisphosphonates in the treatment of FD have been mainly obtained from pamidronate, and data on response to ZA therapy are still limited.

The objectives of this study were: 1) to characterize a cohort of patients with FD/MAS from a single tertiary center in Brazil and 2) to retrospectively evaluate the effects of ZA treatment in this population.

## SUBJECTS AND METHODS

Electronic medical records of patients with FD/MAS evaluated at the Bone Unit of the SARAH Network of Rehabilitation Hospitals in Brasília, Brazil from 2015 to 2020 were retrospectively reviewed. The diagnosis of FD/MAS was established based on clinical and radiological findings, with additional histological evaluation as needed.

Data on age at presentation, sex, symptoms before and after ZA infusion, and extraskeletal complications were collected. Radiographic findings were evaluated to assess the extent and severity of skeletal disease. In addition, we gathered data about medical treatment with ZA, which was administered at 5 mg per infusion, with the timing of subsequent cycles determined by symptoms, severity of disease, and BTMs levels.

Laboratory parameters, including serum levels of creatinine, calcium, phosphate, albumin, 25-hydroxyvitamin D (25 [OH] vitamin D), parathyroid hormone (PTH), total ALP, and serum C-telopeptide of type I collagen (CTX), were also retrieved. Plasma concentrations of calcium, phosphate, albumin, and creatinine were measured using standard laboratory methods; 25 (OH) vitamin D level was analyzed using a chemiluminescent immunoassay; ALP levels were measured by spectrophotometry; and PTH and CTX levels were measured by electrochemiluminescence in a fasting state in the morning. Normal ranges: creatinine 0.6-1.1 mg/dL, calcium 8.5-10.1 mg/dL, phosphate 2.5-4.8 mg/dL in patients age > 18 years, albumin 3.5-5.5 g/dL, 25(OH) vitamin D > 30 ng/mL, PTH 10-69 pg/mL, ALP 42-98UI/L, CTX in premenopausal women < 0.573 ng/mL; < 1.008 ng/mL in postmenopausal women, and < 0.583 ng/mL in men. Normalization rates of ALP and CTX were calculated considering the upper limit of the normal range (ULN) for each one.

The response to ZA treatment was evaluated biochemically based on percentage changes in the maximum levels of BTMs before treatment to the minimum level achieved approximately 6-12 months after treatment, and clinically by relief of symptoms. Bone pain was not quantitatively evaluated using the visual analog scale or other validated questionnaires, and pain response following treatment was categorized as increased, unchanged, improved, or disappeared. Data on the potential adverse effects following ZA infusions were also collected.

Changes in radiological findings in response to ZA therapy were retrieved for patients for whom radiological follow-up was performed. In such cases, radiological examinations after ZA therapy were compared to the previous radiological examinations by an experienced musculoskeletal radiologist. Bone lesion status was categorized as unchanged, improved, or progressive disease if there was increased lytic changes, increase in diameter of the lesions or worsening of bone deformity. Radiological improvement was defined if cortical thickening, progressive filling of destroyed bone and decreased in diameter of the lesion was observed. In addition, data about bone mineral density (BMD) were retrieved for patients who had a dual-energy X-ray absorptiometry (DXA) performed during the follow-up period.

Descriptive statistics were expressed as numbers with percentages, medians, and interquartile ranges (IQR), as appropriate. The non-parametric Mann-Whitney U test was used to compare levels of serum BTMs before and after treatment, as the data did not follow a normal distribution. Spearman correlation was computed to assess the relationship between clinical characteristics (age, time since diagnosis and number of affected skeletal sites) and baseline values of BTMs, and biochemical response to treatment. Statistical significance was set at *P* < 0.05.

This study was approved by the Ethics Committee of the SARAH Network of Rehabilitation Hospitals (Certificate of Presentation for Ethical Appreciation number 31997220.0.0000.0022) and performed in accordance with the tenets of the 1964 Declaration of Helsinki. Informed consent was obtained from all subjects.

## RESULTS

### Subject characteristics

Thirteen patients (two men) with FD were identified, and their general characteristics are summarized in Table 1 . Based on radiological findings, 5 (38%) patients had monostotic and 8 (62%) had polyostotic FD, of whom 3 met the diagnostic criteria for MAS. Two patients with polyostotic FD/MAS experienced precocious puberty, and one of them also exhibited uncontrolled growth hormone (GH) hypersecretion, hyperprolactinemia, and subclinical hyperthyroidism. Craniofacial bones were affected in six (46%) subjects, of whom five presented with variable degrees of craniofacial deformities. The median follow-up duration was 32.9 months (range, 2.2-59.4 months).

**Table 1:** Characteristics of patients with fibrous dysplasia included in the present study

Patient number	Sex	Age at diagnosis	Age at presentation, years	Type	Affected bones	Symptoms / Findings	Biopsy Performed	Baseline ALP		Baseline CTX		Treatment, cumulative dose
								Value (UI/L)	ULN *	Value (ng/dL)	ULN *	
1	F	10	27	Mono	Humerus	Bone pain, previous fracture	No	84	0.86	0.294	0.51	ZA, 5 mg
2	F	36	39	Mono	Lumbar Vertebra (L5)	Asymptomatic	Yes	61	0.62	0.234	0.40	Untreated
3	F	6	36	Poly/MAS	Craniofacial, spine, ribs, humerus, radio, pelvis, bilateral femurs, and tibias bones	Severe craniofacial deformity, exophthalmos, visual impairment, lower limb deformities, previous fractures, bone pain	Yes	1.445	14.75	3.460	6.03	ZA, 20 mg
4	F	9	36	Poly/MAS	Lumbar spine, pelvis, bilateral femurs, left tibia, left fibula,	Bone pain Previous fracture	No	142	1.45	0.744	1.30	ZA, 15 mg
5	M	21	23	Mono	Left acetabulum	Asymptomatic	Yes	104	0.81	0.48	0.82	Untreated
6	F	5	22	Poly	Frontal, temporal, parietal, sphenoidal, zygomatic bones, and occipital.	Bone pain, craniofacial deformity	Yes	205	2.09	0.580	1.01	ZA, 5 mg
7	F	10	31	Poly	Frontal, temporal, parietal, sphenoidal, ethmoidal, zygomatic bones.	Bone pain Craniofacial deformity	No	244	2.50	0.682	1.20	Untreated
8	F	9	34	Poly/MAS	Craniofacial, ribs, left humerus	Craniofacial deformity, café-au-lait macule on trunk and left leg	Yes	223	2.3	1.21	2.11	Untreated
9 **	F	21	61	Poly	Pelvis and left femur	Bone pain, Femur deformity	Yes	129	0.91	1.16	1.15	ZA, 20 mg
10	F	51	52	Mono	Sacrum	Bone pain	Yes	95	0.98	0.741	1.30	ZA, 5 mg
11	M	43	43	Poly	Ribs, lumbar vertebras, sacrum	Bone pain	Yes	-	-	0.522	0.90	ZA, 5 mg
12	F	13	14	Poly	Frontal, ethmoidal, sphenoidal, zygomatic bones involving the orbit	Bone pain Craniofacial deformity	No	129	0.80	1.07	1.90	ZA, 10 mg
13	F	36	37	Mono	Occipital	Mild pain	No	81	0.83	0.175	0.30	Untreated

* ULN: Upper Limit of Normality, expressing the number of times over the upper limit of the normal range;

** Patient with concurrent diagnosis of osteoporosis on long-term ZA therapy. F: female; M: male; ALP: total alkaline phosphatase; CTX: serum C-telopeptide of type I collagen; MAS: McCune-Albright syndrome; ZA: zoledronic acid.

**Figure 1:**
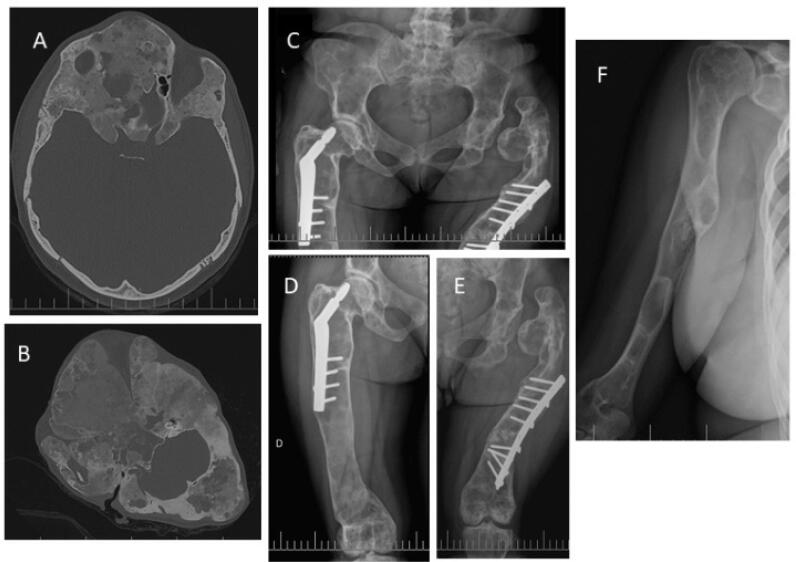
Images of patients with FD showing typical findings. ( **A-B** ) Axial computed tomography images of two different patients with craniofacial involvement, with typical “ground glass” appearance of affected bones and areas of heterogeneity and radiolucency. Severe enlargement of the frontal and occipital regions and thickened cranial base secondary to FD is evident in panel B. ( **C-E** ) Radiographs demonstrate extensive involvement of the pelvis and lower extremities, with severe Shepherd's crook deformity of both femurs, cortical thinning, sclerotic lesions, and “ground-glass” radiolucency. Plate and nail implants were placed in bilateral femurs. ( **F** ) Right humerus radiograph of a woman with monostotic FD showing sclerotic-appearing FD lesions. FD: fibrous dysplasia.

The diagnosis was made solely based on typical radiological findings in five patients, whereas a biopsy was performed for eight subjects. In one patient, molecular analysis of the affected tissue was performed, and the *GNAS* mutation, R201H, was identified. Figure 1 illustrates the typical radiological findings of some patients with FD.

Bone pain of variable intensity was reported by most patients (9, 69%) who used a variety of analgesics, including non-steroidal anti-inflammatory drugs and opioids. Three subjects had prior fractures, and three required mobility assistance with canes or crutches.

At baseline, 8 (62%) patients exhibited serum BTM levels (ALP and/or CTX) above the ULN, four patients had concurrent elevations in levels of ALP and CTX, three patients exhibited an elevated CTX level with ALP in the normal range, and one patient exhibited an elevated ALP level with CTX in the ULN. The median baseline ALP levels were 129 UI/L (IQR 92.2-209.5 UI/L), and the median baseline CTX levels were 0.682 ng/dL (IQR 0.248-1.07 ng/dL). There was a positive correlation between baseline ALP and CTX levels and the number of affected skeletal sites (Spearman r = 0.714, *P =* 0.009, and r = 0.732, *P* = 0.004, respectively), and time since diagnosis (Spearman r = 0.629, *P* = 0.028, and r = 0.584, *P* = 0.036, respectively). All patients exhibited serum phosphate levels within the normal range during the follow-up period.

Craniofacial malignant sarcomatous transformation was reported in one patient with MAS who also exhibited several hyperfunctioning endocrinopathies. She underwent surgical resection of the maxilla and left jaw, followed by adjuvant chemotherapy in another service, without recurrence or additional malignant lesions.

Evaluation of BMD by DXA was available in 4 cases. Two premenopausal women with craniofacial FD presented BMD within the expected range for age (Z-score above −2.0 in lumbar spine, total hip and femoral neck), and DXA scan of a postmenopausal woman with polyostotic FD (P9 – Table 1 ) showed osteoporosis. However, BMD measurements did not include affected skeletal bones in these cases. In a 36-year-old woman with hip involvement (P4 – Table 1 ), total hip BMD was increased (Z-score 3.3) whereas BMD in non-affected lumbar spine was within normal range (Z-score −0,4).

### Medical treatment

Eight (62%) patients were treated with ZA and a 61-year-old woman with polyostotic FD on long-term ZA therapy for a concurrent diagnosis of osteoporosis was excluded from the analysis. The median age of the seven treated patients was 36 years (IQR 24.5-39.5 years), and all of them reported pain in the affected bones. Patients who were not treated (n = 5) either refused ZA due to the potential side effects associated with bisphosphonate therapy (n = 2) or were not considered eligible according to a clinical judgment (n = 3). All but two patients presented with polyostotic disease. The patients received 2.5 courses of ZA on average (range 1-5), and a second cycle was performed a median of 14.2 months (range 5.7-39.2) after the first one.

### Clinical and laboratory response

**Figure 2:**
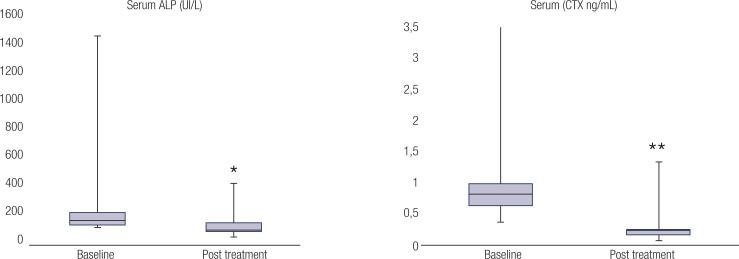
Box plots of medians and interquartile ranges of serum ALP and serum CTX levels of 7 patients with FD before and after ZA treatment. * P = 0.13. ** P = 0.04. ALP: alkaline phosphatase; CTX: C-telopeptide of type I collagen; FD: fibrous dysplasia; ZA: zoledronic acid.

Partial or complete relief of bone pain was reported by all treated patients, except for one whose information about the evolution of pain after ZA therapy was not available in the medical records. Improvement of pain led to a decrease in the frequency of analgesic use, or even discontinuation. In two patients with pelvic and lower limb involvement (P3 and P4, Table 1 ), reduction of bone pain was also associated with improvement of mobility and function, although this was not formally quantified. Following ZA therapy, the median ALP level decreased by 25% (IQR −37.8, −13.0%), but there was no statistical difference in median serum ALP levels after treatment compared to baseline levels (83 UI/L [76.5, 140] *vs.* 135.5 [103.5, 189.3], respectively, *P* = 0.073). Conversely, median CTX levels were significantly lower after treatment than at baseline (0.296 ng/mL, [0.216, 0.298] *vs.* 0.742 ng/mL [0.549, 0.907], respectively, *P* = 0.04), with a change rate of −61.8% (−71, −60%) ( Figure 2 ). Five patients demonstrated a complete biochemical response, with normalization of both BTMs (ALP and CTX). There was no significant correlation between the biochemical response to ZA and clinical characteristics.

### Radiologic findings following ZA therapy

Radiological follow-up was available for three patients treated with ZA. Follow-up imaging was performed using computed tomography (CT) scans in all patients, and one patient also underwent magnetic resonance imaging. One patient had unchanged bone lesion status compared to previous radiological examinations; one patient (P4, Table 1 ) exhibited signs of progression in the pelvis, described as increased lytic changes; and one patient with polyostotic FD/MAS (P3, Table 1 ) showed signs of progression of craniofacial FD. Notably, this patient maintained uncontrolled GH hypersecretion during the follow-up period. Follow-up imaging was performed a median of 27.8 months (range 22-42 months) after the initiation of ZA treatment.

### Adverse events after ZA infusion

Transient fever and/or myalgia within 1-3 days of ZA infusion was reported in 6 out of 7 treated patients, of whom one adolescent also exhibited nausea, vomiting, and transient worsening of headache requiring hospitalization for venous hydration and analgesia.

None of the subjects developed hypocalcemia or renal toxicity. Furthermore, there were no reports of osteonecrosis of the jaw or atypical femur fractures in any patient during the follow-up period.

## DISCUSSION

This study represents the largest single-center review of Brazilian patients with FD and documents the clinical features of 13 patients, including 3 subjects with MAS. Moreover, we retrospectively analyzed the outcomes and complications of ZA therapy in a subgroup of patients who received such treatment. Our data demonstrated that ZA was well tolerated and effective in reducing bone pain and BTMs, notably CTX.

The phenotypic spectrum of our patients was quite variable, ranging from asymptomatic monostotic FD to severely disabling disease affecting almost the entire skeleton. As a mosaic disorder, the phenotypic variability is related to the extent and location of tissues harboring the *GNAS* mutation and reflects the stage of embryogenesis in which the mutation occurs, with earlier mutations resulting in more widespread disease, and those occurring later in development leading to limited disease ( 2 – 4 )

Polyostotic disease was more common (62% of cases), which is in agreement with other reports that suggest polyostotic FD is more prevalent than the monostotic form ( 9 , 10 ). Hyperfunctioning endocrinopathies were observed in two patients with polyostotic FD/MAS. Of note, both experienced precocious puberty, which is a common feature in girls with MAS, affecting approximately 85% of them ( 3 ).

FD/MAS is usually diagnosed on clinical grounds and typical radiological findings; however, histological confirmation may be required, especially for patients with isolated monostotic lesions ( 1 , 2 , 5 ). In our group, eight patients underwent biopsies. Additional molecular analysis of the affected tissue was performed in one patient, and the *GNAS* mutation, R201H, which is the most common *GNAS* pathogenic variant described in the literature, was identified ( 1 , 3 ). However, molecular genetic testing is not routinely required, as it does not typically affect the management of patients with established clinical diagnoses ( 11 ).

Malignant sarcomatous transformations are rare. In our group of patients, one woman with severe polyostotic FD/MAS presented with a history of craniofacial osteosarcoma that was treated at another institution. The prevalence of malignant transformation is difficult to accurately determine due to the rarity of the disease and possible referral bias of tertiary centers, though it seems to be less than 1% ( 12 , 13 ). Prior radiotherapy is a well-known risk factor for malignant transformation. A sudden increase in pain without apparent trauma or a significant expansion of the radiological appearance with cortical destruction should alert the possibility of malignancy ( 3 , 14 ).

Renal phosphate wasting due to overproduction of fibroblast growth factor 23 (FGF-23) in dysplastic lesions may occur in nearly 50% of patients with FD/MAS and can lead to the development of hypophosphatemia and osteomalacia ( 1 , 2 , 15 ). Although serum phosphate levels can fluctuate over the lifespan, hypophosphatemia was not detected in any patient during the follow-up period.

Data about BMD measurement in patients with FD are scarce. In our study, baseline DXA scan was performed in 4 patients, but in only one the evaluation included an affected skeletal bone. In this patient with hip involvement, total hip BMD was increased. A similar finding was described by Chapurlat and cols., suggesting that the typical features of FD and the sclerotic rim within a bone lesion, may interfere with BMD measurements, resulting in an overestimation of BMD ( 16 ).

The natural history of FD is not fully understood. Bone lesions become apparent over the first years of life and tend to expand throughout childhood and adolescence, and the final skeletal burden is usually established by the age of 15 ( 1 , 13 , 17 ). Serum BTMs levels tend to decline throughout adolescence and adulthood, but most subjects persist with values above their age-specific normal range ( 18 ). Age-related radiographic changes have also been described, and FD lesions become more heterogeneous and sclerotic over time ( 1 , 3 ).

Bone pain is a common symptom and can affect the functional status and quality of life. The pathophysiology of bone pain is not completely understood, and its frequency and severity increase with age. Notably, bone pain does not correlate with disease activity or skeletal burden ( 3 , 18 , 19 ). Our findings are in accordance with these concepts, as bone pain was reported by most patients, including those with BTMs within the normal range, and the severity of pain was not related to the extent of skeletal involvement, as patients with polyostotic disease do not necessarily have more severe pain than patients with the monostotic form.

The management of patients with FD/MAS is challenging, and a multidisciplinary team of specialists, including endocrinologists, orthopedic surgeons, craniofacial surgeons, and ophthalmologists is usually required ( 1 , 5 , 11 ). Pharmacological management includes vitamin D repletion (according to specific guidelines), phosphate supplementation, use of active vitamin D analogue if FGF-23 mediated hypophosphatemia is present, and treatment of associated hyperfunctioning endocrinopathies accordingly ( 5 ). However, to date, no treatment has proven to be effective in preventing lesion expansion or improving bone quality.

Bisphosphonates have been used in the treatment of FD because of the increased levels of bone resorption commonly seen in dysplastic bones. Several regimens of bisphosphonates have been tested, but most of the available evidence is derived from observational and uncontrolled studies owing to the rarity of this condition ( 5 , 8 ). The only randomized, double-blind placebo-controlled trial of oral bisphosphonate in FD showed that oral alendronate therapy at 40 mg daily led to a decline in NTX-telopeptides, a biochemical marker of bone resorption, without affecting bone pain, functional status, or radiographic appearance compared to placebo ( 20 ). Therefore, oral bisphosphonates are not recommended for the treatment of bone pain in patients with FD ( 5 ).

Studies with intravenous bisphosphonates, such as pamidronate and ZA, have shown beneficial effects in relieving bone pain and decreasing BTMs, but the response to treatment may be variable and the best treatment regimen and dosing intervals have not yet been established ( 5 ). In the study of Wang and cols, partial or complete remission of bone pain was reported in 64% of patients after bisphosphonate therapy, and ALP levels decreased by 22.7% and 34.1% in the pamidronate and ZA groups, respectively ( 7 ). In a case series of 5 patients with FD, of whom 4 were treated with pamidronate and one received ZA, improvement of bone pain was reported by all subjects ( 21 ). In an open study of patients treated with pamidronate, pain intensity decreased by 41% after the first cycle of pamidronate in all patients with pain at baseline (n = 44), and by 69% after several treatment cycles. Furthermore, ALP, osteocalcin and urinary CTX significantly reduced after therapy ( 6 ). In another open study of 41 patients treated with bisphosphonates, a complete clinical and biochemical response to treatment, with relief of symptoms and normalization of ALP levels, were described in 24 of 30 patients with polyostotic FD (80%), and in 4 of 11 patients with MAS (36%) ( 22 ). Although most patients respond favorably, Thomsen and cols., did not find any clear relief of symptoms in patients treated with bisphosphonates ( 9 ), and ZA did not result in substantial improvement of bone pain and BTMs in patients who were unresponsive to long-term pamidronate treatment ( 16 ).

Based on these data, ZA was offered to our patients who presented with FD-related bone pain and/or elevation of BTMs. We observed that ZA effectively reduced bone pain, although it was not quantitatively measured using the visual analog pain scale or other validated pain questionnaires. In addition, we demonstrated a beneficial effect of ZA therapy on the reduction in BTMs levels, which are commonly used as markers of metabolic disease activity. At baseline, most patients (62%) exhibited BTMs (ALP and/or CTX) above the ULN, of whom three patients had elevated CTX with ALP levels within the normal range. Baseline ALP and CTX levels also positively correlated with the number of affected bones, which was consistent with previous studies ( 7 , 22 ). In the subgroup of patients treated with ZA, serum ALP and CTX levels decreased by 25% and 61.8%, respectively, and median CTX values were significantly lower after treatment than at baseline. These findings suggest that CTX may be a more sensitive marker to monitor disease activity and response to treatment than ALP. However, the value of CTX in the evaluation of FD requires further investigation, as it has not been frequently assessed in other reports ( 7 , 16 , 23 ). Considering that changes in BTMs relative to therapy were evaluated within 6-12 months after ZA infusion, we believe that our findings reflect the response to therapy rather than the well-known age-related decline in bone turnover ( 18 ).

The possible effect of intravenous bisphosphonates in improving bone quality or preventing bone expansion is controversial. Radiographic response, described as filling of osteolytic lesions, and cortical thickening, has been described in some reports with pamidronate or ZA, although it was not a consistent finding ( 16 , 23 , 24 ). In our study, the number of patients with radiological follow-up was small (n = 3), but no benefit in the imaging aspect was observed. Notably, progression of craniofacial FD was detected in one patient with uncontrolled GH hypersecretion, which is a well-known condition associated with craniofacial expansion and poor prognosis ( 2 , 5 ).

Considering the natural history of the disease, in which FD lesions increase in childhood and their metabolic activity decreases over time after the final skeletal burden is achieved, it is plausible that strategies of treatment to prevent the development or expansion of FD lesions would need to be targeted during childhood and initiated soon after the development of FD becomes apparent ( 1 ). Florenzano and cols. did not find any benefits of bisphosphonate therapy on the progression of disease burden in children. However, regimens of bisphosphonates used were variable, and the formulation and doses used by these subjects were not specified ( 18 ). Recently, Kumar and cols. evaluated the effects of ZA treatment in 12 children with FD and reported significant improvement in radiological findings following therapy, suggesting that the drug may be effective when administered early ( 25 ). However, the absence of a control group precludes a definitive conclusion, and prospective trials with larger sample sizes are required to clarify this issue.

The acute phase response (fever, headache, myalgia, malaise), which usually occurs within 24-48 hours following infusion, is a well-known adverse event of ZA therapy. In patients with osteoporosis, it occurs in approximately 18% of cases after the first infusion; however, the incidence rate decreases in subsequent infusions ( 26 ). In our cohort, most patients (five out of seven) experienced acute phase reactions following the first ZA infusion, but no serious side effects have been reported to date, including osteonecrosis of the jaw, which seems to be more common among patients with FD (5.4%) than in patients with osteoporosis treated with bisphosphonates ( 27 ).

Our study had certain limitations. First, it was based on a retrospective analysis, and missing data were unavoidable. In addition, it included a heterogeneous group of patients, and the timing and doses of ZA were variable. Moreover, as previously mentioned, pain and functional status were not formally evaluated using quantitative validated tools, and a placebo effect could not be excluded. However, given the rarity of the disease and paucity of available data on its treatment with ZA, our findings provide a substantial contribution to the current literature, and reinforce the role of ZA as an option in the medical management of FD-related bone pain.

In summary, the clinical manifestations of FD were heterogeneous, and some patients presented a debilitating course. ZA therapy was generally well tolerated and provided beneficial effects on the relief of bone pain and a reduction of bone turnover, although without radiological benefits. Prospective controlled studies are still required to assess the efficacy and long-term safety of ZA therapy in patients with FD.
